# Population genetic structure of a recent insect invasion: a gall midge, *Asynapta groverae* (Diptera: Cecidomyiidae) in South Korea since the first outbreak in 2008

**DOI:** 10.1038/s41598-023-29782-8

**Published:** 2023-02-16

**Authors:** Ji Hyoun Kang, Daseul Ham, Sung Hwan Park, Jeong Mi Hwang, Sun-Jae Park, Min Jeong Baek, Yeon Jae Bae

**Affiliations:** 1 Korean Entomological Institute, Korea University, Seoul, 02841 Korea; 2 National Institute of Biological Resources, Incheon, 22689 Korea; 3 Division of Environmental Science and Ecological Engineering, College of Life Sciences & Biotechnology, Korea University, Seoul, 02841 Korea

**Keywords:** Population genetics, Entomology, Invasive species, Molecular ecology

## Abstract

Outbreaks of *Asynapta groverae*, an invasive mycophagous gall midge, in South Korea have been repeatedly reported since the first occurrence in 2008. This species is a nuisance to residents owing to its mass emergence from newly built and furnished apartments. Here, the levels of genetic diversity, divergence, and structure of invasive *A. groverae* populations were investigated to understand their ability to survive in novel locations. Population genetic analyses were performed on seven invasive populations, including the first outbreak, sporadically emerged, and two laboratory-isolated (quarantined) populations, using the mitochondrial *COI* sequences and the ten novel microsatellite markers developed in this study. Non-indigenous *A. groverae* managed to maintain their populations for 12 years despite decreased genetic polymorphisms resulting from multiple incidences of founder effects by a small number of colonists. Additionally, the advantageous sustainability of *A. groverae* in the particle boards from which they emerge suggests that human-mediated dispersal is plausible, which may allow for the successful spread or invasion of *A. groverae* to new locations. This study is one of the few examples to demonstrate that an insect species successfully invaded new regions despite exhibiting decreased genetic diversity that was maintained for a decade. These findings indicate that the high genetic diversity of the initial founding population and asexual reproduction would contribute to the successful invasion of *A. groverae* in novel environments.

## Introduction

Globally, invasive species in non-native ranges are among the leading threats to biodiversity, ecosystems, and industries^1–5^. Recent climate change events, including global warming, have facilitated the survival and adaptation of newly invaded populations to novel local habitats, which were previously not the ecological niche of the invasive species^6,7^. Additionally, globalization in recent years has increased human activities (such as trade and travel), which have further contributed to the spread of invasive species^8,9^. After the invasion, alien species may either not survive owing to unfit environmental conditions or successfully colonize new habitats and expand. Established populations of alien species can be a threat to the community if they exhibit predation and/or competition with endemic species, leading to the disturbance of the whole ecosystem. Several cases of invasive species-induced ecological and economic damages have been reported. The identification of the processes involved in the establishment, dispersal route, and spread of alien species is challenging^10,11^.

**Figure 1:**
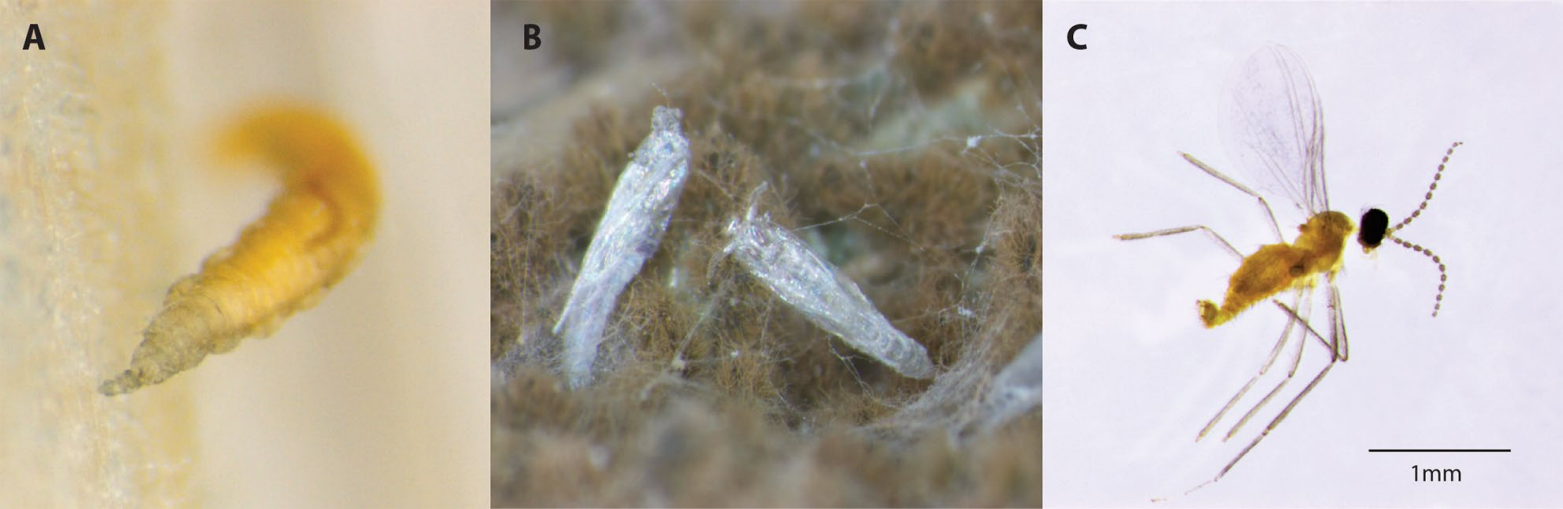
Larva (**A**)^12^, pupal case (**B**), and an adult male (**C**) of *Asynapta groverae.*

**Figure 2:**
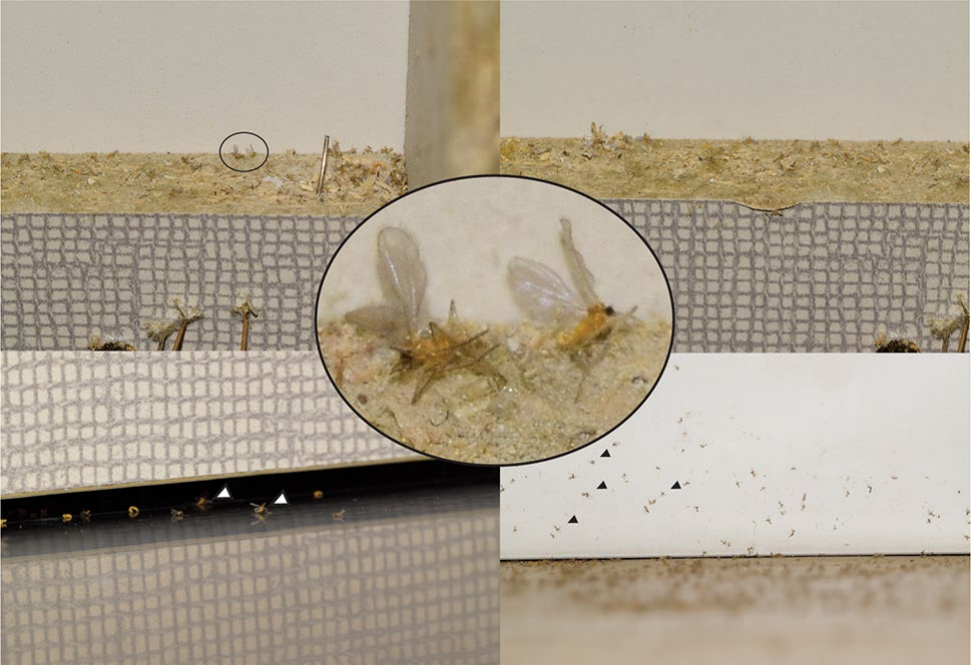
Outbreaks of *Asynapta groverae* in particle boards. Adult individuals are shown in the black circle and black and white. Not all individuals are marked.

The first outbreak of *Asynapta groverae* Jiang and Bu (Porricondylinae, Cecidomyiidae), a mycophagous gall midge, in South Korea was recorded in 2008^12^ (see Fig. 1). This small gall midge was first described in Madhya Pradesh, India as *Dicerura indica*^13^. The small gall midge was later renamed *Asynapta indica* by recombining the genus name but subsequently named as *Asynapta groverae* by Jiang and Bu^14^. The first outbreak of *A. groverae* in newly furnished apartments in South Korea was reported in 2008^12,15^. Since 2008, sporadic outbreaks have been reported across the country, especially during the spring and summer seasons^16^ (Fig. 2). *Asynapta groverae*, which was not previously recorded in this region, is the only known species of the genus *Asynapta* in South Korea. Globally, 49 *Asynapta* species have been recorded^12,17^. The first morphological description of the larval and pupal stages of *A. groverae* has been recently published^16^.

Limited studies have reported the outbreak pattern and ecological characteristics of *A. groverae*^12,15^. The adult lifespan of *A. groverae* is 3–4 days, that is, no longer than one week, whereas the larval period is approximately 2–3 weeks long. Thus, the generation time from egg to adult is approximately 3–4 weeks under optimal environmental conditions, such as temperature and humidity^15^. The sole source of *A. groverae* outbreaks in Korea is the particle boards, which are used for manufacturing furniture and are a place of inhabitation and emergence for larvae and adults (Fig. 2). Particle boards are manufactured from lumber waste products and used for the construction of new furniture for apartments. Additionally, particle boards can be manufactured using mycelium-based composites, which are synthetic construction materials that use natural fungal growth. Thus, particle boards are susceptible to infections from all fungi^18^. Most *A. groverae* individuals were found on the fungus-infected part of the particle boards. The larvae of *A. groverae* feed on fungi^12,15^. Thus, particle boards are a rich source of fungal food for *A. groverae* larvae*.* Outbreaks of *A. groverae* in the form of adults, larvae, and pupae from newly built and furnished apartments are a nuisance to residents (Fig. 2)^15^ owing to the increased amount of insect biomass during the emerging period in indoor conditions. However, *A. groverae* do not serve as allergens or pathogens to humans^15^. Complete eradication of the *A. groverae* has not been successful as a few individuals were found even after one month post-pest control^15^.

Although continuous outbreaks of *A. groverae* have been reported during the last decade^12^, the ecological characteristics (such as voltinism, generation time, and life history), origin, and dispersal route of *A. groverae* have not been elucidated. Furthermore, the *A. groverae* genetic attributes, such as the level of genetic diversity, population differentiation and structure, and temporal stability of gene pools, have not been reported. The genetic diversity level and population structure are strongly associated with the successful colonization of invasive species into novel habitats^19–22^. Moreover, the genetic diversity of alien species is low during the initial invasion as the invasion is mediated by a few colonists, representing a small fraction of the source population (founder effect)^23,24^. Small and isolated populations are prone to genetic drift owing to the bottleneck or founder effects^25^. A previous study reported that a genetic bottleneck facilitated the successful invasion of *Linepithema humile*, an Argentine ant species^26^. The loss of genetic diversity due to a population bottleneck in the invasive Argentine ant population decreases intraspecific aggression by forming interspecifically dominant supercolonies^26^. In contrast, a relatively high genetic diversity of the founder population has been reported in cases of simultaneous or independent introduction of multiple source populations^27,28^. For example, the high genetic diversity of the invading wasp population resulted in successful invasion into the introduced ranges^29^. Decreased or elevated genetic variations in the introduced population could lead to the successful invasion of a broad suite of taxa according to circumstances.

Population genetic and phylogeographic analyses can reveal invasion processes, such as the origin, invasive history, introductory routes, dispersal/expansion patterns, and temporal and spatial genetic variation of invasive populations, which provide essential information for effective and efficient invasive species monitoring and control^30–32^. For example, population genetic analyses of the pinewood nematode *Bursaphelenchus xylophilus* revealed several characteristics of invasive processes from the native range of North America to Europe^33^. Low levels of genetic polymorphisms detected among the introduced populations of *B. xylophilus* suggested a single event of invasion during the invasion process, which contributed to a small effective population size^33^. Moreover, a large effect of genetic drift in invasive populations was suggested based on the strong genetic structure of native populations^33^. Population dynamics of introduced species, such as range expansion patterns^34^, invasive population size^19^, and ecological niche^20^, have been recognized in diverse taxa.

Microsatellites, which are single sequence repeats, have been commonly used in population genetic studies on various insect taxa to identify genotypic diversity^35^, population genetic differentiation^36^, biological invasion^37–39^, host range expansion^40^, sexual selection^41^, ecological characteristics^42^, and speciation^43^. However, most microsatellites serve as species-specific markers. Hence, cross-species amplification of markers is limited to the genus level in most cases^44,45^. Most microsatellite markers have been developed for economic damage-causing species belonging to the family Cecidomyiidae^46–50^. Currently, microsatellite markers have not been identified for *Asynapta* species.

This study, for the first time, identified genome-wide microsatellites for *Asynapta* species and investigated the genetic diversity and structure of seven invasive *A. grovera*e populations, including first reported (2008), sporadically emerged (2018–2020), and two laboratory-isolated populations. Population genetic analyses were also performed using the mitochondrial *COI* sequences. The analysis of spatio-temporal genetic patterns for 12 years since the first outbreak will provide useful insights into the successful invasion of *A. groverae* in South Korea from a population genetics perspective.

## Results

### mtDNA diversity, haplotype network, and population structure

**Figure 3:**
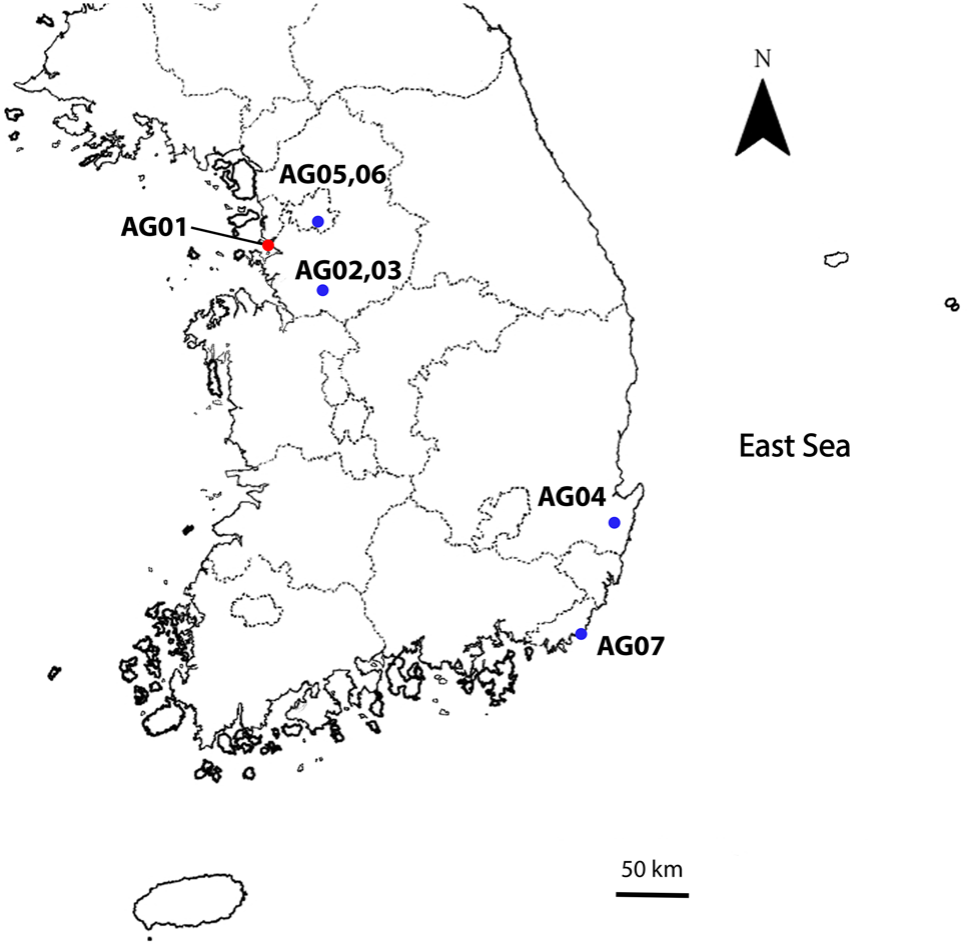
Map of sampling location from where 121 specimens of *Asynapta groverae* were collected in South Korea. Site of the first outbreaks of *A. groverae* (AG01) is shown in the red circle.

Among the 121 *A. groverae* specimens from seven populations, the sequence of *COI* (648 bp) (Table 1; Fig. 3) had only 13 polymorphic nucleotide positions (nine parsimonious informative sites and four singletons). In total, 12 haplotypes (H1–H12) with low levels of genetic divergence were identified among the 121 *A. groverae* temporal samples collected in 2008 and between 2018 and 2020 (GenBank accession numbers OK561689–OK561809) (Table 2). The *N*_H_ values ranged from 1 to 6 for each population. AG04 exhibited the highest *N*_H_ value (six), whereas AG06 (an isolated population originating from the AG02 population) exhibited an *N*_H_ value of 1 (Table 3). The overall *h* and π values for all seven populations were 0.465 and 0.002, respectively (Table 3).

**Table 1:** Information of sampling sites, population code, type of population (e.g., natural and isolated), latitude/longitude, and sampling year for *Asynapta groverae* populations in South Korea.

Population code	Sampling localities	Type of population	Latitude	Longitude	Year
AG01	Songdo	Natural	37° 22′ 40″ N	126° 38′ 53″ E	2008
AG02	Dongtan A	Natural	37° 11′ 26″ N	127° 07′ 40″ E	2018
AG03	Dongtan B	Natural	37° 11′ 26″ N	127° 07′ 40″ E	2018
AG04	Pohang	Natural	35° 57′ 11″ N	129° 24′ 38″ E	2019
AG05	Laboratory	Isolated	37° 11′ 26″ N	127° 07′ 40″ E	2019
AG06	Laboratory	Isolated	37° 11′ 26″ N	127° 07′ 40″ E	2019
AG07	Gijang	Natural	35° 16′ 09″ N	129° 13′ 36″ E	2020

**Table 2:** Characteristics of newly developed microsatellite loci for *Asynapta groverae*.

Locus	GenBank accession Nr	Forward primer1 (5′–3′)	Repeat motif	Locus size (bp)	*N*_A_	PIC	*H*_E_	*H*_O_
Ag_156	OK545742	F: AAGATGAATTGGACCAGC R: AACAAGTAGCGAGCGATC	(GA)^17^	209	31	0.949	0.955	0.759
Ag_273	OK545743	F: TTCTCACAGCTGGATATTAC R: GAACGATTACAGATTGTGAC	(CT)^19^	162	19	0.844	0.861	0.799
Ag_279	OK545744	F: GTGTATGTGCTGTGAACA R: TGTTGCGCTTATTCTCTC	(AG)^19^	171	12	0.581	0.619	0.332
Ag_292	OK545745	F: TAATTGATGTTAGTCGGTCC R: GAGCGACCGATTCTTATC	(TC)^19^	174	29	0.825	0.846	0.755
Ag_295	OK545746	F: CAACGCTTGCATATCATATC R: GGTTGTTGGTCGGTTATT	(GA)^20^	202	22	0.890	0.902	0.864
Ag_339	OK545747	F: AATGTACAGTCACCGTGAAT R: TTCATGTAGTTGACCAGTTG	(AG)^19^	179	27	0.851	0.871	0.359
Ag_392	OK545748	F: ATAGAATGACAATCGCCG R: GCAATGATACGATGATGAAG	(AG)^17^	299	15	0.715	0.750	0.264
Ag_423	OK545749	F: AATGGACGATGACAACAG R: GATATTCCAGCGACCATC	(GGC)^12^	147	9	0.545	0.588	0.479
Ag_3276	OK545750	F: CTGGTAATTATGGAACGTTG R: CTGAGGTGATGTATATTGGT	(CAG)^13^	176	15	0.831	0.850	0.726
Ag_3281	OK545751	F: CACGAACATCAACATCAAC R: TGTAACCGTTGATACAGTC	(CAT)^12^	176	11	0.823	0.844	0.583

*N*_*A*_, Total number of alleles detected; PIC, Polymorphic information content; *H*_E_, Expected heterozygosity; *H*_O_, Observed heterozygosity.

**Table 3:** Summary of genetic diversity statistics in seven populations of *Asynapta groverae* in South Korea at both the mitochondrial *COI* region and ten novel microsatellite loci.

Population	Year	mtDNA						Microsatellites						
		*N*	*N*_H_	HR	PH	*h*	*π*	*N*	*N*_A_	AR	*H*_E_	*H*_O_	*F*_IS_	H–W tests (*P*)
AG01	2008	18	4	2.544	0	0.543	0.001	20	8.7	7.099	0.745	0.486	0.355	**
AG02	2018	20	3	1.453	0	0.279	0	20	9	7.078	0.775	0.637	0.182	**
AG03	2018	19	5	2.526	0	0.386	0.001	20	8.7	6.908	0.757	0.608	0.201	**
AG04	2019	18	6	4.137	2	0.791	0.003	20	9.9	7.74	0.827	0.716	0.138	**
AG05	2019	18	3	1.877	0	0.464	0.004	20	7.9	6.436	0.773	0.611	0.214	–
AG06	2019	12	1	0	0	0	0	20	5.1	4.418	0.653	0.554	0.155	**
AG07	2020	16	3	2.25	3	0.35	0.001	20	9.2	6.991	0.73	0.53	0.279	–
Total	–	121	12	3.8195	4	0.465	0.002	140	8.4	7.895	0.807	0.576	0.218^†^	**

mtDNA, Mitochondrial DNA; *N*, Sample size; *N*_H_, Number of haplotypes; HR, Haplotype richness; PH, Number of private haplotypes; *h*, Haplotype diversity, π, Nucleotide diversity; *N*_A_, Observed mean number of alleles across eight loci; AR, Allelic richness; *H*_E_, Expected heterozygosity; *H*_O_, Observed heterozygosity; *F*_*IS*_, Observed inbreeding coefficient; H–W tests (*P*), *P* value for multi-locus tests for Hardy–Weinberg equilibrium, average of each *F*_IS_^†^

**Figure 4:**
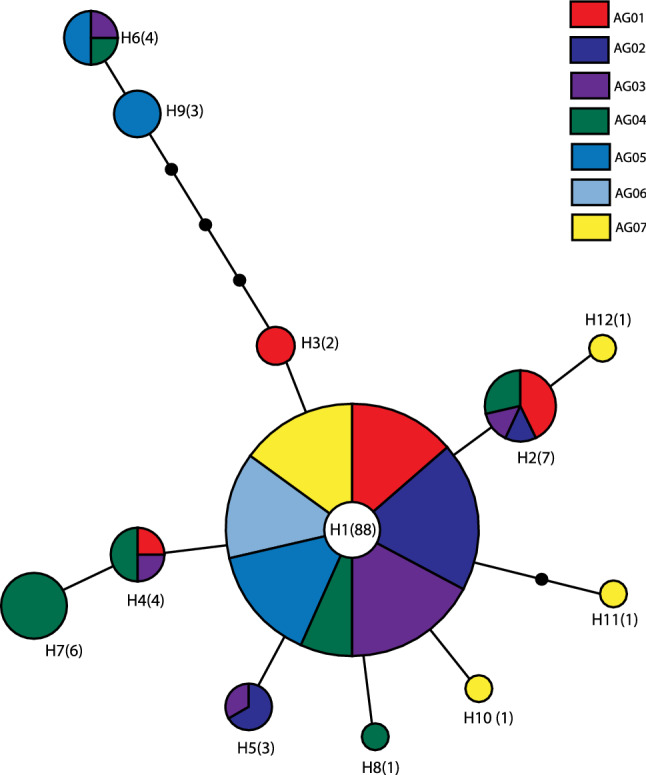
Haplotype network of *COI* sequences from seven populations of *Asynapta groverae*. The area of the circle is proportional to the individual numbers of the respective haplotype. Numbers shown in parentheses represent the number of individuals belonging to a haplotype. Each line in the network represents a single mutational step between haplotypes irrespective of its length. Different colors denote different populations.

A haplotype network for *COI* sequences from seven *A. groverae* populations exhibited a star-like pattern. The most common haplotype (H1) was in the center of the network surrounded by other haplotypes (Fig. 4). The major haplotype (H1, 73% of all specimens) located in the most internal position in the network (presumably ancestral) was identified from the specimens collected from all seven populations, including AG01 (specimen from the first outbreak population in 2008 in South Korea). The haplotypes at the edge of the network with only a few mutational steps (< 4) separated from the major haplotypes were observed in the individuals from the populations collected between 2018 and 2020.

Of the 21 tests for pairwise *F*_*ST*_ values calculated among the seven populations, six exhibited significant mitochondrial genetic differentiation (− 0.004 to 0.351) (Table 4). The highest value of genetic differentiation was identified during the comparison between a laboratory-reared population (AG06) and a recently collected population in 2019 (AG04). Comparison of AG01 with AG03 and AG02 revealed no genetic differentiation (Table 4). The samples were collected in different years (10-year difference) from geographically close sampling sites (Fig. 3). Moderate levels of genetic differentiation (*F*_ST_ > 0.1) were mostly observed during comparisons with the AG04 population (Table 4).

**Table 4:** Pairwise genetic differentiation (*F*_ST_) based on mitochondrial *COI* region sequences (below diagonal) and ten microsatellite loci genotypes (above diagonal) for seven populations of *Asynapta groverae* from South Korea.

	AG01	AG02	AG03	AG04	AG05	AG06	AG07
AG01	–	**0.040**	**0.047**	**0.019**	**0.085**	**0.112**	**0.082**
AG02	0.036	–	**0.008**	**0.013**	**0.066**	**0.085**	**0.010**
AG03	− 0.004	− 0.035	–	**0.028**	**0.087**	**0.105**	**0.107**
AG04	**0.115**	**0.254**	**0.187**	–	**0.063**	**0.089**	**0.083**
AG05	0.029	0.040	0.002	**0.167**	–	**0.141**	**0.037**
AG06	0.142	0.031	0.045	**0.351**	0.122	–	**0.158**
AG07	0.025	0.015	0.027	**0.212**	0.014	0.041	

Significant values (*P* < 0.05) are in bold.

AMOVA revealed the lack of significant genetic structures between the following population pairs: temporal grouping, the first outbreak population in 2008 and sporadically emerged populations between 2018 and 2020; spatial grouping, north and south populations; habitat grouping, natural and isolated populations. Most of the variance occurred among populations irrespective of the group (*F*_ST_) (Table S1).

### Development and characterization of novel microsatellite markers

High-throughput sequencing generated 76,509,371 reads with 1,887,386 scaffolds containing di-, tri-, and tetra-nucleotide repeat motifs (average length = 173 bp). The number of perfect microsatellite sequences, which were suitable for primer design, was 106,753 containing 72,317 di- (68%), 32,214 tri- (30%), and 1819 tetra-nucleotides (2%) repeat motifs. Of the 20 initially screened markers, 10 polymorphic microsatellite markers were successfully amplified with stable and reproducible amplicon patterns and distinct peaks in capillary electrophoresis (Table 2). The number of alleles for each locus ranged from 9 to 31. The mean polymorphic information content (PIC) across loci was 0.7856, representing highly polymorphic markers. The highest PICs (0.949) were observed at the AG_156 locus (Table 2). *H*_O_ per locus ranged from 0.264 to 0.864 (mean = 0.8053), whereas *H*_E_ ranged from 0.588 to 0.955 (mean = 0.5939). In total, 181 alleles (average = 18) were amplified from these markers within and across the populations and used for subsequent population genetic analysis.

### Microsatellite diversity and population structure

The within-population genetic diversity was relatively high with the *H*_E_ values in the range of 0.653–0.827. The highest *H*_E_ and *H*_O_ values were observed in the AG04 population (Table 3). The mean *N*_A_ per population was 8.4 (range: 5.1–9.9) (Table 3). The AR level ranged from 4.418 to 7.099. The lowest AR was observed in one of the laboratory populations (AG06), which was similar to the observations with the *COI* locus. All populations, except AG05 and AG07, significantly deviated from the HWE (Table 3). The level of inbreeding (*F*_IS_) ranged from 0.138 to 0.355. The highest *F*_IS_ value was observed in AG01, which was obtained from the first outbreak population in 2008.

All pairwise *F*_ST_ values calculated among the seven populations were significant (p < 0.01) and were in the range of 0.008–0.141 after Bonferroni correction (Table 4). These findings suggest that the invaded populations were genetically differentiated at the microsatellite loci analyzed.

**Figure 5:**
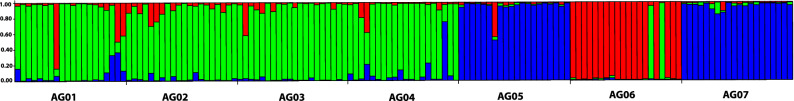
Population genetic structure of the seven *Asynapta groverae* populations determined using a Bayesian population assignment test with STRUCTURE based on ten microsatellite loci. All individuals are shown along the X-axis. The Y-axis denotes the probability of that individual belonging to each of the genetic clusters. The most likely number of genetic clusters after Delta K Evannos’ correction is 3 (*K* = 3). The population of all individuals is indicated below the plot.

STRUCTURE analysis of ten microsatellite genotypes represented the three most likely distinct genetic clusters (K = 3; *Δ K* = 200.35) from seven *A. groverae* populations (Fig. 5). The bar plot reveals that seven populations were barely admixed although a proportion of all three genetic clusters was identified in all populations (Fig. 5). The first outbreak population in 2008 (AG01) and other populations collected from 2018 to 2019 (AG02, 03, 04) were predominantly represented by a specific genetic cluster (in green). The lack of significant genetic differentiation among the four populations was further supported using FCA^51^ (Supplement Information, Fig. S1). Both AG05 (rearing population) and AG07 (collected in 2020 from Gijang, located distantly from the first outbreak site) exhibited similar structures (in blue). The weak genetic structure between AG05 and AG07 was further supported using FCA and *F*_ST_ values (Tables 4 and S1). AG06, the other isolated population, was the only population in which most individuals were assigned to the other genetic cluster (in red).

AMOVA of microsatellites revealed the absence of genetic structures for all tested groupings (similar to observations with the *COI* locus) (Supplementary Information, Table S1). Significant variance was observed only within populations (Table S1).

## Discussion

This study analyzed the spatio-temporal genetic population structure and genetic diversity levels in the invasive populations of *A. groverae*, a mycophagous gall midge, in South Korea using mitochondrial *COI* sequences and a novel set of ten nuclear microsatellite markers. Population genetic analyses were performed to gain insights into the successful invasion and persistence of *A. groverae* over the last 12 years from an ecological/evolutionary genetic perspective. The status of *A. groverae* as an invasive species in South Korea should be carefully verified before examining its origin, dispersal route, and population dynamics. Invasive species can be referred to as “a species that arrives (often with human assistance) in a habitat it had not previously occupied, then establishes population and spreads autonomously”^52^. India is reported to be the original habitat of *A. groverae*. The first outbreak of *A. groverae* was recorded in Korea in the Songdo region in 2008. Accurate species identification based on a combined analysis of morphological and genetic data is critical to study newly invasive species^53^. Specimens of larvae, pupa, and adults were carefully examined in the current study based on the various taxonomical keys following the first description of this species^14^, our previous studies on *A. groverae*^12,16^, other taxonomical investigations on asynaptine species^54,55^ and Cecidomyiidae^56^ (Supplementary Information, Figs. S2 and S3). Taxonomical examinations on the specimens in the different developmental stages further supported that *A. groverae* has never been reported in Korea before 2008. Unfortunately, however, molecular-based species identification (i.e. DNA barcoding) could not be applicable for *A. groverae* since only a single *COI* sequence is available from GenBank database for the genus *Asynapta* and its species was not even identified (*Asynapta* sp.)^57^.

Since 2008, several sporadic outbreaks (emergence) have been reported during the last 12 years. Furthermore, field investigations using sticky traps around the sites where outbreaks occurred and manufacturers of the particle boards were located did not reveal any evidence of *A. groverae* habitation in Korea^15^. All outbreaks occurred only in newly built apartments with new furniture manufactured from particle boards. No outbreaks from other habitats have been reported in Korea^15^. Although the possibility of the previous existence of *A. groverae* Jiang and Bu (Diptera: Cecidomyiidae) in Korea cannot be completely ruled out, this small gall midge can be considered a recently colonized invasive species through anthropogenic activities, such as trade and transport of wood materials from the native range for its dispersal.

The level of genetic polymorphisms in this invasive gall midge population in Korea was higher than that in other invasive cecidomyiids^28^. *Obolodiplosis robiniae*, a North American gall midge species, was first detected in 2004 and has spread extensively throughout China. The mean *H*_E_ value of 22 invasive populations (20 individuals per population) at 14 microsatellite loci was 0.5346 in China (invasive population), whereas that of US native populations was 0.6606^28^. Although a direct comparison between the two members of cecidomyiids is not possible, the* H*_E_ value of *A. groverae* ranged from 0.653 to 0.827, which is markedly higher than that of *O. robiniae*. Low genetic diversity is often expected in the initial phase of invasion as limited numbers of colonists have typically been introduced as a founding population^23,24^. However, relatively high genetic polymorphisms in the founding population (AG01) of *A. groverae* may contribute to overcoming the genetic hurdle of early invasive processes^28^.

Invasive colonies/populations of this non-indigenous gall midge species have not flourished or completely disappeared over the last 12 years in Korea even though decreased genetic diversity is known to be detrimental to the persistence and adaptation of populations in novel environments^58–60^. The findings of this study demonstrated that the gene pool of the populations has not markedly changed for more than 10 years. The initial levels of HR and AR in AG02 and AG03 (collected in 2018) were maintained or slightly decreased when compared with those in AG01 (collected in 2008). In addition to decreased levels of genetic diversity, the level of genetic divergence was low as evidenced by some mutational steps from the ancestral haplotypes (Fig. 4). Only three genetic clusters were identified for all populations (Fig. 5) and no temporal genetic differentiation was detected using AMOVA (Supplementary Information, Table S1). However, the invasive populations exhibited detectable genetic differentiation, especially at microsatellites. This suggests that they are genetically divergent owing to the genetic drift effects during independent invasion events within South Korea. Outbreaks have been consistently reported in 2011, 2012, and 2014–2018 across the country since 2008^28^.

Thus, the repeated successful invasion of this species for 12 years is an area of active research. The simplest explanation involves repeated multiple invasions (introductions) of *A. groverae* from the source population. The increased migration rate of the introduced population by multiple repeated invasions would mitigate decreased genetic diversity and inbreeding, which can contribute to successful invasion^61^. In particular, the genetic diversity of the AG04 population was slightly higher than that of the founding population (AG01). Wood materials used to produce particle boards, which are the source of all known outbreaks and *A. groverae* habitat, were imported from several countries in East Asia^15^. Multiple populations of *A. groverae* with a small number of individuals may have been repeatedly introduced from their native ranges during the last decades. If founding individuals have multiple source populations, increased genetic diversity and marked genetic divergence can be expected^27,28^. However, genetic polymorphisms have not increased in succeeding *A. groverae* populations, except AG04, in this study. The haplotypes found in 2019 and 2020 were not markedly different from those found in 2008 (Fig. 4). The genetic divergence levels in distantly located populations (AG04 and AG 07 populations collected in 2019 and 2020, respectively) were lower than those in the first reported population (AG01). Thus, repeated introductions of *A. groverae* to Korea from abroad might be a less likely hypothesis.

Alternatively, a founding population of *A. groverae*, which was introduced before or around 2008, has been maintained as a single invasion source during the last 12 years. The ability of *A. groverae* to survive and continue its life cycles on particle boards would facilitate the spread and persistence of the population. Particle boards can be stored in one place and distributed to manufacture furniture in newly built apartments in different regions. This study, for the first time, reported that the life cycle of *A. groverae* continued from the particle board even after one year in a separate box under laboratory conditions (Figs. 1, 2). Thus, this study is one of the few examples that demonstrated that invasive populations can be maintained as small populations with diminished genetic diversity for a prolonged duration without additional invasion events. This indicates that increasing genetic variation from multiple invasions and the marked population growth of invasive species in a new ecosystem might not always be a priori conditions for the successful colonization of non-native species into novel environments. This phenomenon has been argued as a ‘genetic dilemma’ in invasive species in which bottlenecked populations become invasive species despite having a low level of genetic diversity and low adaptive ability to new environments^59^. Introduced populations with low genetic variation can overcome their reduced evolutionary potential and reproductive fitness by asexual or self-fertilizing mode of reproduction, which leads to a high reproductive rate and removes deleterious alleles that cause inbreeding depression^61–63^. Although the reproductive strategy in *A. groverae* was not identified in this study, *A. groverae* may become a successful invader if asexual reproduction is possible in this species, irrespective of the level of genetic variability that would affect evolutionary potential and reproductive success.

Paedogenesis, which is parthenogenetic reproduction at the larval or pupal stages, has been reported in several Cecidomyiidae species since its first discovery in *Miastor metraloas* by Wagner^64–66^. Laval paedogenic reproduction has been identified in *Heteropeza pygmaea* Winnertz and *Mycophila speyeri* (Barnes) of Cecidomyiidae^64,67–69^. A recent molecular phylogeny analysis of Cecidomyiidae revealed that these two lineages appear to be basal to *Asynaptini* (to which *A. groverae* belongs)^70^. Paedogenesis, which is the larval reproductive life cycle, is also reported to be related to the optimal use of food patches, which leads to a short generation time in insects^71^. If fungal food resources are sufficient, larvae can continue their paedogenetic life. The larvae will start developing and undergoing metamorphosis under unfavorable conditions and subsequently fly away in search of new fungal sources^72,73^. Most *A. groverae* larvae and adults were found at the fungi-rich part in the particle boards in the outbreak population, as well as in the two isolated populations (AG05 and 06) after one year in the sealed box. Thus, particle boards can provide a rich fungal food source for *A. groverae* and ensure asexual reproduction with high propagation rates, which would allow for the continuous establishment of invading populations.

Although a particular genotype (shown in green in Fig. 5) was predominant in the source population (AG02), the two reared populations (AG05 and AG06) were almost assigned to the other two genotypes (shown as blue and red, respectively). Furthermore, all seven populations were almost individually assigned to one genetic group. These results indicate that the settlement of the newly invaded populations is a stochastic event involving a random process. Most *A. groverae* outbreak populations were small and isolated from particle boards. Many Cecidomyiidae species are poor flyers, and their long-distance dispersal must be assisted by other external factors, such as wind^74–76^. Thus, settlements of *A. groverae* are formed by multiple founder events and the resulting genetic drift effects of decreased diversity.

The isolated populations emerging from the AG02 population may suggest that the dispersal of *A. groverae* through the transportation of particle boards is plausible. In this study, particle boards in which *A. groverae* emerged in 2018 from one site (AG02) were collected and maintained for one year in a separate box at room temperature without any treatment. *A. groverae* could survive as larvae or eggs on the particle boards and successfully reproduce even after one year, which indicates the high sustainability of *A. groverae*. The remarkable ability of *A. groverae* to survive in the particle boards may further explain the reason for the sporadic appearance of *A. groverae* during the last 12 years, as well as for the non-decline of the population due to fitness reduction resulting from decreased genetic diversity and inbreeding. Similar to AG05, the most recent naturally emerging population (AG07) from the Gijang region also formed one genetic cluster (shown in blue). Thus, if particle boards function as a potential vehicle for dispersing *A. groverae* populations into new environments, human activity-mediated dispersal could result in founder events or bottlenecking during the invasion processes of this species.

The findings of this study provide useful insights into the successful colonization of the recently introduced *A. groverae* species and establishment of multiple invasive populations from the founding population during the early invasion processes. A combination of high genetic diversity within the founding population and the potential capability of asexual reproduction enabled this insect to become a successful invader. Furthermore, this study suggests that successful invasions and the subsequent dispersal of invasive species can be facilitated by anthropogenic activities, which can contribute to the sustainability of invasive species in new environments.

## Methods

### Sampling

Five populations of *A. groverae* adults were collected from newly furnished apartments in 2008, 2019, and 2020 (Table 1; Figs. 1, 2, 3). All specimens were carefully examined with bright-field and phase contrast microscopy (Olympus BX50, Japan). Species was diagnosed by various morphological key characteristics such as antenna and antero-ventral papillae present, spatula absent for larvae, and vein of wing, the number of flagellomeres, shape of genitalia, and pattern of seta under gonostylus claw for adult male and vein of wing, the number of flagellomeres, and shape of abdomen terminalia for adult female^12,14,16,56^ (Figs. S2, S3). The AG01 population was collected in 2008 when the first outbreak of *A. groverae* was reported in South Korea. These specimens were stored in the KU collection at Korea University. Other populations were collected from recent outbreaks from the particle boards of the newly furnished apartments in 2018 and 2019 (Fig. 3). However, the AG05 and AG06 populations were isolated (quarantined) at the laboratory at Korea University to test for the possibility of particle boards as a dispersal route of *A. groverae* (Fig. 3). Two isolated (quarantined) populations, originating from AG02, were collected in 2018. The particle boards from which the AG02 population was collected were maintained in a separate box in the laboratory for one year at room temperature. Separate particle boards were maintained for the AG05 and AG06 populations. New individuals that emerged from each population (each particle board) in 2019 were used for this study. All the specimens were preserved in 100% ethanol until analysis.

### Mitochondrial DNA sequencing

Total genomic DNA was isolated from the thorax of the specimens using the DNeasy blood & tissue kit (Qiagen, USA). The mitochondrial DNA (mtDNA) *COI* sequences (648 bp) were amplified using the universal primers LCO1490 (5′-GGT CAA ATC ATA AAG ATA TTG G-3′) and HCO2198 (5′-TAA ACT TCA GGG TGA CCA AAA AAT CA-3′)^77^. Polymerase chain reaction (PCR) was performed in a 20-μL reaction volume under the following conditions: an initial denaturation at 94 ℃ for 1 min, followed by 35 cycles of 94 ℃ for 30 s, 50–52 ℃ for 30 s, and 72 ℃ for 1–2 min; and a final extension step at 72 ℃ for 7 min. The amplicons were visualized on 1.5% agarose gels using UV light, purified using exonuclease I and shrimp alkaline phosphatase (New England BioLabs, USA), and sequenced using an ABI PRISM 3130xl Genetic Analyzer (Applied Biosystems, USA) by Macrogen INC Sequencing (Korea). *COI* sequences of *A. groverae* obtained in this study were deposited in GenBank (accession no: OK561689 and OK561809).

### Development of novel microsatellite markers

Total genomic DNA was extracted from the thorax of adults (AG05) using the DNeasy blood & tissue kit (Qiagen, USA), following the manufacturer’s instructions. The integrity, concentration, and purity of the DNA were assessed using a Bioanalyzer 2100 (Agilent, USA). The sheared genomic DNA was processed to construct an Illumina paired-end library using a NxSeq^®^ EZ-UltraLow DNA library kit. Next, paired-end reads (150 bp) were generated using the Illumina NextSeq500 platform (Illumina, USA). For assembly, sequencing errors were removed using the error correction module of SOAPec (ver. 2.02)^78^. Sequencing adaptors were trimmed using Skewer (ver. 0.2.2)^79^. Genome assembly was performed using SOAPdenovo2 (ver. 2.04-r240). To identify reliable assemblies, short reads were remapped to assembled sequences using GapCloser (ver. 1.12). Only assembled scaffolds were retained for microsatellite marker identification. In total, 1,887,386 scaffolds with an average length of 173 bp were obtained. Microsatellite sequences with 2–6 repeat motifs were identified using MISA^80^. The candidate loci were validated using PCR with primers designed using Primer 3^81^. The primers were designed based on the following parameters: length, 20–26 nucleotides; annealing temperature, 51–54 ℃; product size, 100–300 bp. A set of 20 candidate microsatellite primer pairs was initially tested for polymorphisms. Among these, ten microsatellite markers were chosen. The GenBank accession numbers for the ten loci are listed in Table 2.

### Microsatellite genotyping

One of each primer pair for the novel microsatellite markers was labeled with the fluorescent dye 5′-FAM. PCR was performed in a 20-μL reaction volume containing 30 ng DNA, 10 mM dNTPs, 100 nM of each primer, and 5 U HANLAB Taq polymerase (HANLAB, Korea) in an ABI 2720 Thermocycler (Applied Biosystems, USA). The PCR conditions were as follows: an initial denaturation at 95 ℃ for 2 min, followed by 35 cycles of 95 ℃ for 20 s, 50 ℃ for 30 s, and 72 ℃ for 30 s, and a final extension step at 72 ℃ for 5 min. The amplicons were electrophoresed on an ABI 3730x automated DNA sequencer and fragment sizes were analyzed using the GENEMAPPER software v. 5. (Applied Biosystems, USA).

### Population genetic analyses

Mitochondrial diversity indices, including the number of haplotypes (*N*_H_), haplotype richness (HR), haplotype diversity (*h)*, and nucleotide diversity (π), were estimated for each population and the entire pooled population using AREQUIN v.3.5.^82^. HR was estimated to correct for unequal sample size using the refraction method with CONTRIB v1.02^83^. The haplotype network was obtained using HAPSTAR v0.7^84^. Microsatellite diversity was assessed based on the mean number of alleles per locus (*N*_A_), allelic richness (AR), expected (*H*_E_) and observed heterozygosity (*H*_O_), and observed inbreeding coefficient (*F*_*IS*_) using GENEPOP v4.0^85^ and FSTAT v.2.9.3.2^86^. The presence of null alleles was assessed using MICROCHECKER v. 2.2.3^87^ with 1000 randomizations at 95% confidence level. Multi-locus tests for Hardy-Weinberg equilibrium (HWE) and null allele frequency at each locus were estimated using GENEPOP v4.0. To examine population differentiation, exact tests for population differentiation and the calculation of population pairwise *F*_*ST*_ values were performed for both markers using GENEPOP v4.0. Pairwise population comparisons were performed for significance using Bonferroni correction. Temporal and spatial genetic structures of *A. groverae* among the seven populations were assessed using hierarchical analysis of molecular variance (AMOVA) implemented in ARLEQUIN v.3.5. Seven populations were assigned to two temporal groups (collected in 2008 (AG01) and 2018–2020 (AG02–07)). Two biogeographic regions (north (N: AG01, 02, 03, 05, 06) and south (S: AG04, 07)) were assigned according to geographical distance irrespective of the collection year. Additionally, two isolated (quarantined) populations were assigned to the isolated group (I: AG05, 06) and natural population (N: AG01, 02, 03, 04, 07). The total molecular variance was partitioned among groups (Fct = ‘inter-group’ genetic variation), populations within groups (Fsc = ‘intra-group’ genetic variation), and within populations (Fst = ‘inter-population’) (Supplementary Information, Table S1).

The population genetic structures of *A. groverae* populations were examined using the Bayesian clustering algorithms implemented in STRUCTURE (v. 2.3.1)^88^ under a model of admixed ancestry among populations and correlated allele frequencies. Likelihood scores were calculated using the genetic clusters for each K value from 1 to 7 with 100,000 burn-in steps, followed by 1,000,000 Markov chain Monte Carlo iterations. Subsequently, the most likely K value was estimated using the web-based tool Structure Harvester (http://taylor0.biology.ucla.edu/structureHarvester/) based on the rate of change in the log probability data between successive K values. Furthermore, factorial correspondence analysis (FCA) based on genetic relationships among individuals with multi-locus genotypes was performed using GENETIX v4.5.2^89^

## Supplementary Information

Supplementary Information 1.

Supplementary Information 2.

Supplementary Information 3.

Supplementary Information 4.

## Data Availability

The datasets generated and/or analysed during the current study are available in GenBank under the accession numbers OK545742-OK545751, OK561689–OK561809 (https://ncbi.nlm.nih.gov/nuccore).
