# Supplementary material for: Population genetic structure of a recent insect invasion: a gall midge, Asynapta groverae (Diptera: Cecidomyiidae) in South Korea since the first outbreak in 2008
Source: Sci Rep. 2023 Feb 16;13:2812. doi: 10.1038/s41598-023-29782-8 (PMC9935521; doi:10.1038/s41598-023-29782-8)
Supplement: Supplementary file 1 — Supplementary Information 1. [file 41598_2023_29782_MOESM1_ESM.pdf]

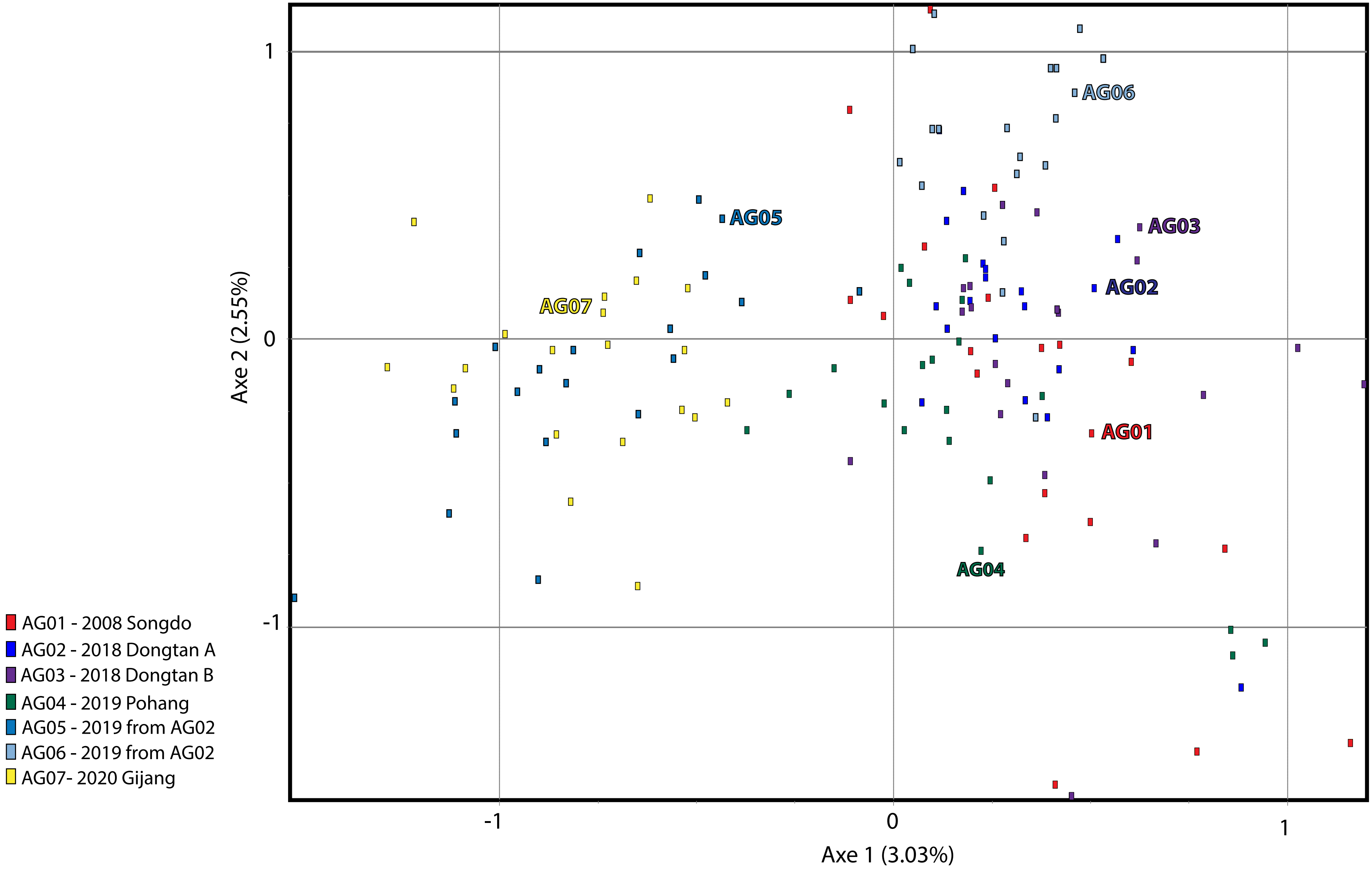

Figure S1. Results of factorial correspondence analysis of microsatellite allelic variation. Different colors indicate the members of the corresponding populations.
