# Supplementary figures and images for: Population genetic structure of a recent insect invasion: a gall midge, Asynapta groverae (Diptera: Cecidomyiidae) in South Korea since the first outbreak in 2008
Source: Sci Rep. 2023 Feb 16;13:2812. doi: 10.1038/s41598-023-29782-8 (PMC9935521; doi:10.1038/s41598-023-29782-8)

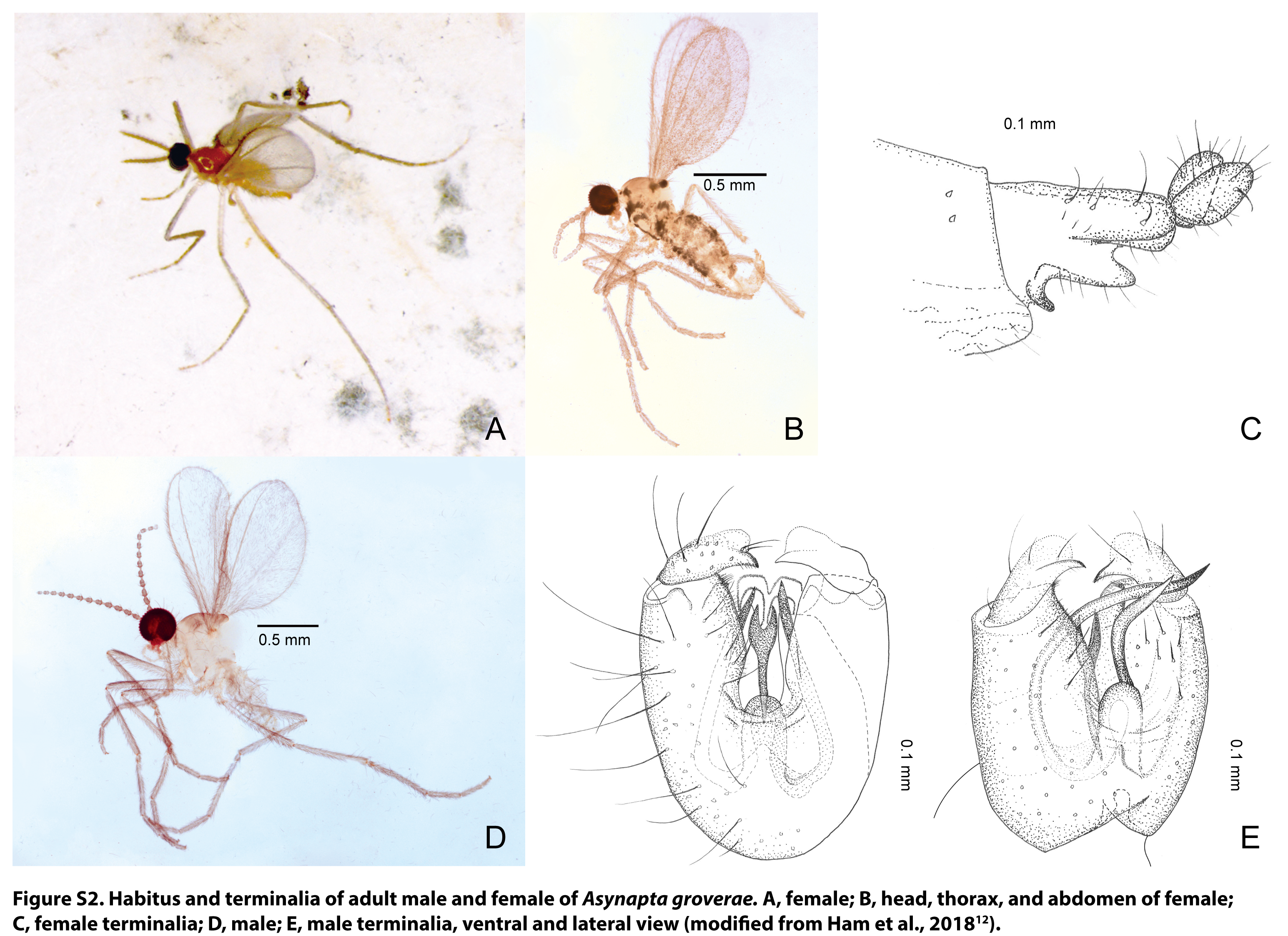

Supplement: Supplementary file 2 — Supplementary Information 2. [file 41598_2023_29782_MOESM2_ESM.tif]

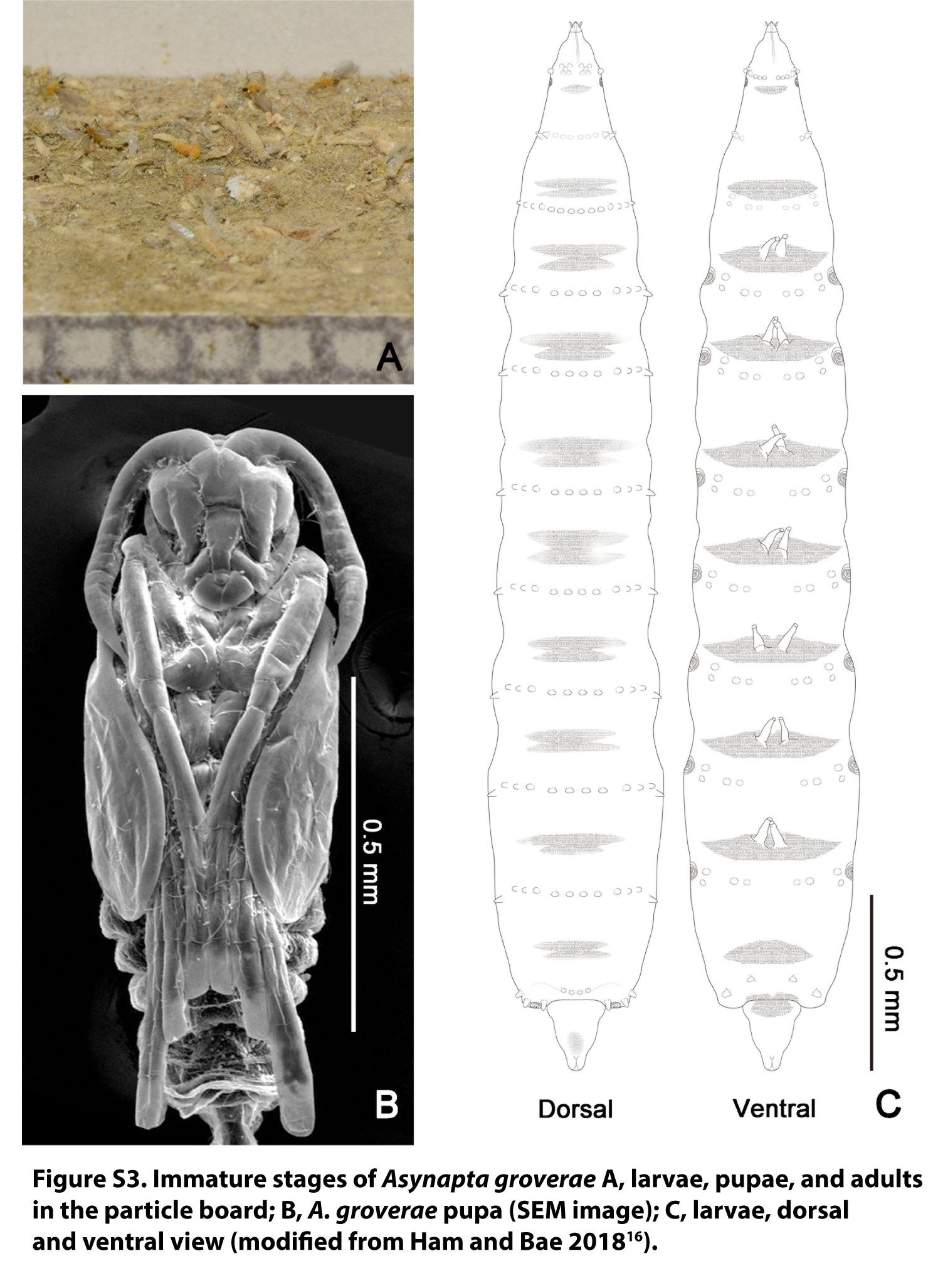

Supplement: Supplementary file 3 — Supplementary Information 3. [file 41598_2023_29782_MOESM3_ESM.tif]
