# Supplementary material for: Population genetic structure of a recent insect invasion: a gall midge, Asynapta groverae (Diptera: Cecidomyiidae) in South Korea since the first outbreak in 2008
Source: Sci Rep. 2023 Feb 16;13:2812. doi: 10.1038/s41598-023-29782-8 (PMC9935521; doi:10.1038/s41598-023-29782-8)
Supplement: Supplementary file 4 — Supplementary Information 4. [file 41598_2023_29782_MOESM4_ESM.docx]

**Table S1.** Analysis of molecular variance (AMOVA) of *Asynapta groverae* in South Korea. Significant *P-*values (*P* < 0.05) are indicated in bold.

|  | Grouping | Source of variation | | d.f. | Sum of squares | Variance components | % Variation | Fixation index |
| --- | --- | --- | --- | --- | --- | --- | --- | --- |
| mtDNA | Temporal  (First vs. sporadic outbreaks) | | Among groups | 1 | 0.506 | -0.053 | -9.69 | FCT: -0.097 |
|  |  | | Among population within groups | 5 | 10.192 | 0.090 | 16.57 | **FSC: 0.151** |
|  |  | | Within populations | 114 | 0.505 | 0.505 | 93.12 | **FST: 0.069** |
|  | Two spatial (N vs. S) | | Among groups | 1 | 2.234 | 0.011 | 1.88 | FCT: 0.019 |
|  |  | | Among population within groups | 5 | 8.464 | 0.069 | 11.80 | **FSC: 0.120** |
|  |  | | Within populations | 114 | 57.525 | 0.505 | 86.32 | **FST: 0.137** |
|  | Natural vs. Isolated | | Among groups | 1 | 2.644 | 0.025 | 4.14 | FCT: 0.041 |
|  |  | | Among population within groups | 5 | 8.054 | 0.064 | 10.71 | **FSC: 0.112** |
|  |  | | Within populations | 114 | 57.525 | 0.505 ­ | 85.15 | **FST:0.149** |
| microsatellites | Temporal  (First vs. sporadic outbreaks­­) | | Among groups | 1 | 4.697 | - 0.099 | -3.77 | FCT: -0.038 |
|  |  | | Among population within groups | 4 | 45.145 | 0.220 | 8.36 | **FSC: 0.081** |
|  |  | | Withing populations | 234 | 586.000 | 2.504 | 95.40 | **FST: 0.046** |
|  | Two spatial regions (N vs. S) | | Among groups | 1 | 8.999 | -0.029 | -1.09 | FCT: -0.011 |
|  |  | | Among population within groups | 5 | 61.315 | 0.244 | 9.06 | **FSC: 0.090** |
|  |  | | Withing populations | 273 | 678.125 | 2.484 | 92.03 | **FST: 0.080** |
|  | Natural vs. Isolated | | Among groups | 1 | 13.539 | 0.020 | 0.71 | FCT: 0.007 |
|  |  | | Among population within groups | 5 | 56.675 | 0.221 | 8.12 | **FSC: 0.082** |
|  |  | | Withing populations | 273 | 678.125 | 2.484 | 91.17 | **FST: 0.088** |
